# Assessment of the Frequency of Sweetened Beverages Consumption among Adults in Poland

**DOI:** 10.3390/ijerph18137029

**Published:** 2021-06-30

**Authors:** Agnieszka Piekara, Małgorzata Krzywonos

**Affiliations:** 1 Department of Bioprocess Engineering, Wroclaw University of Economics and Business, Komandorska 118-120, 53-345 Wrocław, Poland; 2 Department of Process Management, Wroclaw University of Economics and Business, Komandorska 118-120, 53-345 Wrocław, Poland; malgorzata.krzywonos@ue.wroc.pl

**Keywords:** sugar sweetened beverages, sugar sweetened beverages consumption, obesity, sugar tax

## Abstract

*Background*: in the context of the ongoing obesity epidemic and increase in cases of metabolic disorders among the population, it is significant, from the health, nutritional, and economic point of view, to a look at the habits of sugar-sweetened beverage (SSB) consumption of adults in Poland. This study aims to determine the sweetened beverages, which are the most popular and most frequently consumed by Polish consumers in correlation with sex, age, income, and education of the studied group of adults. *Methods*: The survey study was based on the Computer-Assisted Web Interview (CAWI) and was conducted on a representative sample of 500 adult Poles, within the period from May to June 2020. The fundamental stage of the survey included the question, which asked the respondents to assess the frequency of consuming selected eleven sweetened or unsweetened beverages. Socio-economic characteristics of the respondents were considered when evaluating whether these factors aligned with the behaviors regarding the frequency of beverage consumption. *Results*: The consumption of SSBs tends to decrease along with consumer’s age. A decrease, which could be associated with level of consumers’ education level and correlation with income could not be verified. Adults drink sugar sweetened beverages less often than younger consumers, and women drink them less often than men. *Conclusions*: Identification of the frequency of sugar-sweetened beverage intake can indicate directions for further national or regional action against the spread of obesity. Moreover, attention should be drawn to young males who consume beverages abundant in high sugar most often.

## 1. Introduction

Currently, more than 100 countries produce sugar. About 85% of the global production is sugarcane-based. In the last 20 years, world sugar consumption has increased consistently (aside from for a slight decrease in 2018 and 2019) from 123.5 MM tonnes to 177.8 MM tonnes, forecast in 2020 [1,2]. The potential impact of the COVID-19 pandemic on the global economy and sugar production and sugar consumption remains highly uncertain.

Sugar is used to provide sweetness and is an adaptable and probably irreplaceable functional ingredient in food. In addition to providing sweetness, sugar also has other applications such as balancing acidity, adding bulk or prevent spoilage, among other things. Beyond that, sugar is also used to produce medications or bioplastics [3,4]. Therefore, sugar-sweetened beverages (SSB) are one of the product groups in which sugar and other sweetening agents are a necessity. According to the U.S. Department of Agriculture, sugar-sweetened beverages are liquids, which have been sweetened using a variety of forms of added sugar such as brown sugar, corn sweetener, corn syrup, dextrose, fructose, glucose, high-fructose corn syrup, honey, lactose, malt syrup, maltose, molasses, raw sugar, and sucrose. Regular soda (the non-sugar free type), fruit, sports, and energy drinks, sweetened waters as well as beverages such as tea and coffee which have been sweetened with sugars are all examples of sugar-sweetened beverages [5,6]. Both, the sweetening agents as well as the addition of coloring agents may intensify the negative effects of SSB consumption on human health [7].

The economic interests of the sugar industry is at odds with the WHO recommendations warning about the ongoing obesity epidemic or even pandemic. One of the primary causes of such a situation is the high consumption of sugar in the diet. In addition, it was proven that obesity and overweight have further negative health consequences [8,9,10,11]. The obesity problem affects Poland to a large extent. According to a survey conducted by the National Institute of Public Health-National Institute of Hygiene (NIZP-PZH) in October 2020 over 54.5% of Poles aged 18 and more suffered from being overweight or obese [12]. These results were slightly above the average for European Union countries in 2014, which amounted to 51.6% (35.7% of people suffering from being overweight and 15.95 suffering from obesity, respectively) [12]. Along with an increase in the number of overweight people who suffer from obesity-caused illnesses, there is an increase in the social costs related to treating them. According to the 2010 WHO data, the expenses connected with treating obesity complications among adults in Europe absorb between 6 and 10% of the budgets intended for health protection [13,14].

In Poland, the total expenditures on health care amounted to 16.95 B euros in the year 2017, taking into account the direct costs of treating obesity exceeded 1.11 B euros, while if we consider the indirect expenses (related to lost worktime, health benefits, and premature deaths) even 3.3 B euros [15]. In France, for example, the social cost of overweight and obese individuals in 2012 was as estimated to be as high as 20 B euros (about 1% of GDP) equivalent to the social cost of tobacco consumption [16]. Moreover, it is significant that many people are unaware of the WHO guidelines for sugar intake [17].

Lately, the most attention is given to the consumption of SSB among children and young people [18,19,20,21,22,23]. However, it is equally important, from the health, nutritional, and economic point of view, to take a look at the habits of adults. An additional reason to look at adult consumption is that many adults are parents and influence the consumption patterns of their children. This study aimed to determine the most popular and the most frequently consumed sweetened beverages among Polish consumers in relation to sex, age, income, and education of the studied group of adults. That’s why the research question sounds: what kind of sweetened beverages is the most popular among the Polish consumers? What kind of sweetened beverages do they prefer the most in the term of frequency of SSB consumption? Both the socioeconomic characteristics of the respondents and the main sugar-related concerns were taken under consideration when evaluating beverage consumption. A comparison of the consumption of the 11 main sweetened and unsweetened beverages was conducted, taking into account the sex, age, education, and wealth of the respondents.

## 2. Materials and Methods

The survey study was based on the Computer-Assisted Web Interview (CAWI) and was conducted within the period from May to June 2020 on a representative sample of 500 adult Poles. The quota sampling method was chosen to ensure that structure of the respondents was consistent with the distribution of most of the demographic variables for Polish residents (sex, age). Information obtained from respondents was held in confidence. Data was collected via an anonymous, self-administered on-line questionnaire, and information such as name, e-mail address, postal address or any other personal information were not requested. Validation was conducted by pre-testing on a group of 10 consumers (diverse in terms of age, education, wealth, and gender) to limit the risk related to the potential lack of understanding of questions by respondents and rejecting irrelevant statements. The questionnaire developed by the authors was addressed to adults (more than 18 years of age) Poles. There were no criteria other than the amounts provided for in the sample distribution. The invitation was sent to 73,217 people and the study was conducted until the assumed sample (*n* = 500) was achieved. Only four questionnaires were not filled out completely and for that reason they were rejected. The study was carried out after the first wave of the Covid-19 pandemic when restrictions were partially lifted.

Descriptive statistics and crosstabs were used for the analysis of participants’ characteristics. Dependencies were confirmed using the chi-square (*p* ≤ 0.05), which allows for confirmation of the statistical significance of the compared variables. The results of the survey were subjected to statistical analysis using the statistical software packages aPQStat (version 1.6.4, PQStat Software, Poznań, Poland) and Statistica (version 12, StatSoft Poland, Krakow, Poland).

Consuming tap water is possible, and many local authorities encourage citizens to do it. However, many Polish citizens don’t have a habit of doing so, and it is perceived as unsafe by some consumers. The 11 main beverages were selected based on: market analysis (evaluation of what is available for sale) and the literature on what kind of beverages were analyzed.

Their caloric value ranges per 100 mL of the beverages selected for evaluation (based on the label declarations of products available for sale) were as follows: still mineral water (0.0 kcal), carbonated mineral water (0.0 kcal), flavored water (up to 52.0 kcal), fruit juices (20.0–50.0 kcal), vegetable juices (17–21 kcal), fruit nectar (26–64 kcal), still fruit beverages (12–42 kcal), carbonated fruit beverages (up to 48 kcal), carbonated beverages e.g., coke/cola or soda type (up to 44.0 kcal), energy drinks (up to 47 kcal), sports/isotonic drinks (up to 46 kcal). The caloric value of each beverage may vary and it depends on the composition e.g., in the case of juices or nectars it depends on the type of fruit used in their production.

## 3. Results

### 3.1. Characteristics of the Respondents

A total of 500 respondents took part in the study (Table 1). The gender division in the research group was as follows: 52% of the respondents are male, and 48% of the respondents-female. The 18–29 age range was represented in the study by 87 respondents. The respondents aged 30–44 were represented by a total of 149 respondents, whereas the 45–59 age range comprised 116 respondents. As many as 148 of the respondents were over the age of 60.

### 3.2. Consumption Results

The fundamental stage of the survey included the question, which asked the respondents to assess the frequency of their consumption of eleven particular beverages. This assessment was based on the 6-point scale (never, sporadically, few times a month, 1–2 a week, 3–4 times a week, every day or almost every day), which refers to the consumption of selected sweetened beverages and water (Table S1).

The relation between the gender and frequency of consumption of selected drinks was analyzed (Table S1). According to the conducted research, women drink more mineral water on an everyday basis than men (73.3% of the female respondents and 59.6% male respondents). Non-carbonated fruit beverages are consumed less frequently by men than by women (chi-square independence test confirmed the statistical significance, *p* ≤ 0.05). The opposite can be observed in the case of carbonated beverages and carbonated fruit beverages. Fruit juices are consumed regularly at least once a week by a comparable percentage of men and women. However, women drink more juices on an everyday basis (17.2%), whereas the % of men drinking juices amounts to 13.8%. The lack of a noticeable difference in regular consumption (minimum once a week) was observed in the context of flavored waters and carbonated fruit beverages. The analysis of the answers provided by the respondents and referring to carbonated beverages of the ‘coke’ and soda type and carbonated fruit beverages, which usually are the most abundant in caloric sweetening agents, found that men are likely to consume them more than women on an everyday or nearly everyday basis. Even though these results are not statistically significant (Table S1) the importance of this cannot be ignored. A relatively higher percentage of women stated that they do not drink energy drinks (36.7% female respondents, 25.4% male respondents) or isotonic drinks (women 43.3%, men 26.5%). More men than women drink such beverages regularly (minimum once a week)—this trend is particularly noticeable in the case of energy drinks in reference to consuming them every day or 3–4 times a week. 

For the purpose of the study, it was necessary to analyze consumption frequency in contrast to the structure of respondents’ age. The respondents, regardless of their age, stated that most often they drink noncarbonated, mineral water on an everyday basis (66%). Carbonated mineral water was consumed by 30.2% of the respondents. Statistical significance was proven in the case of: flavored water (*p*-value 0.001), fruit nectars (*p*-value 0.044), carbonated fruit beverages (*p*-value 0.013), carbonated beverages (*p*-value 0.034), and energy drinks (*p*-value 0.023) as well as isotonic drinks (*p*-value 0.003) (Table S2).

Few Poles drink flavored water on an everyday basis (Table S2). Flavored water, which may contain aromas or caloric or non-caloric sweetening agents, is consumed most often and regularly, with a minimum frequency of once a week, by the respondents aged 30–44 (45.0% of the respondents from said age group). The highest percentage of respondents declaring that they never drink beverages of this type can be found among the elderly respondents (over 60 years).

Based on the conducted research, it can be concluded that fruit nectars were consumed most regularly (minimum once a week) by the respondents in the 18–29 age group. Older respondents drink fruit nectars quite rarely in comparison with other age groups. The highest percentage of respondents who do not drink nectars or drink them sporadically can be found in the over 60 years old age group.

**Figure 1:**
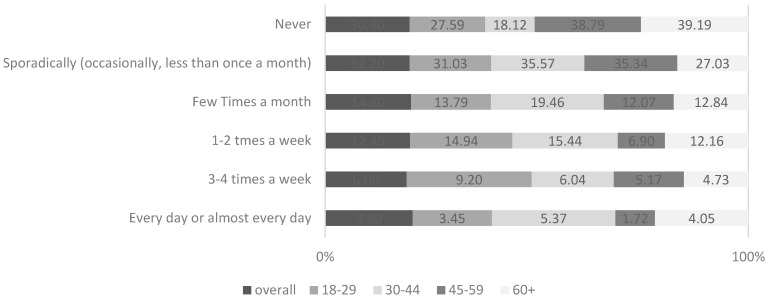
The age of the respondents versus the frequency of energy drinks consumption *n* = 500.

The younger consumers decidedly more often choose carbonated fruit beverages. The percentages of young consumers who declare that they drink such beverages regularly, a minimum once a week, in both age ranges are comparable. On an everyday basis, the younger respondents choose them twice as often as the respondents over the age of 45. Respondents who are over the age of 45 consume carbonated beverages significantly more often. The highest percentage of the respondents who consume carbonated drinks of the ‘Coke’ or soda type regularly, minimum once a week, are from the 18–29 and 30–44 age groups, respectively. According to the survey, the lowest percentage of respondents who regularly drink carbonated beverages was observed in the 45–59 age group. The Chi-square independence test has also indicated statistical significance between the frequency of consumption of energy (Figure 1) and isotonic drinks and the age of the consumers (Table S2).

Energy drinks are most often consumed by respondents from the 30–44 age group. Moreover, based on the responses provided by the remaining age groups, it can be observed that the percentage of people over 60 years of age who do not drink energy drinks is the highest. The consumption of such beverages among the respondents aged 45–59 is relatively low. Poles below the age of 45 drink energy drinks more often than older consumers.

The following observations can be made based on the analysis of the energy and isotonic drinks consumption, taking into account the age structure of the study group:

- in the 45–59 age range as well as in the oldest age group, isotonic drinks are consumed relatively rarely 48.3% and 39.9% of the respondents, respectively, declared that they never drink such beverages;
- the highest percentage of people who regularly consume such beverages (minimum once a week) can be observed in the 30–44 (23.4%) and 18–29 (16.0%) age groups.

**Figure 2:**
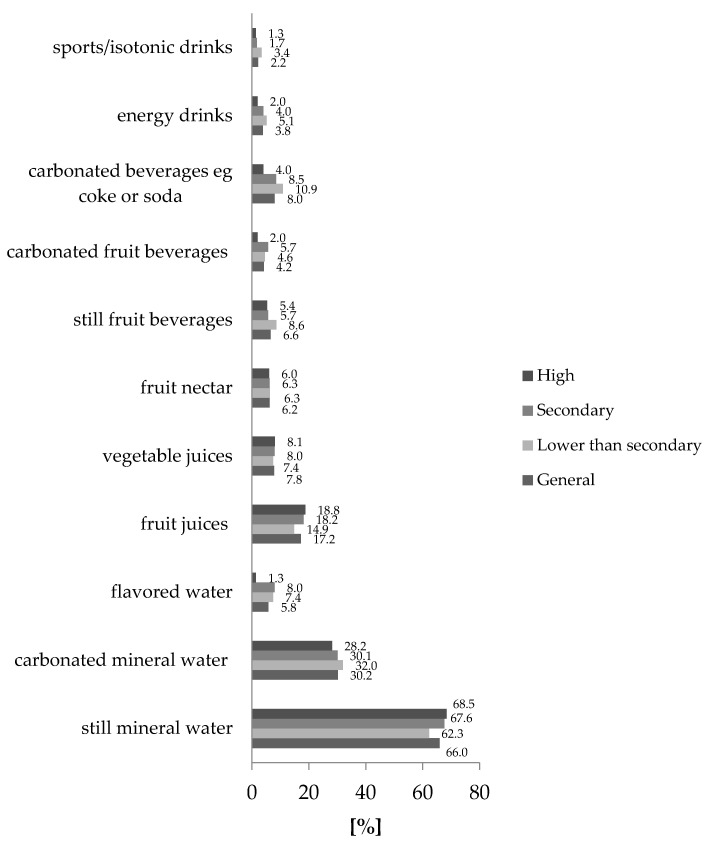
Relationship between drinks consumed on an everyday basis and education.

Regardless of education, a similar percentage of respondents declared they consume still mineral water on an everyday basis. Analysis of the obtained results referring to carbonated mineral water, fruit nectars, and fruit, as well as vegetable juices, did not showany significant differences in the percentage distribution of the responses (Figure 2).

The results showed that fruit juices are consumed less often by people who completed lower than secondary education 14.0%. Whereas, in the case of the people with secondary or higher education, the percentage amounts to, respectively, 18.2% and 18.8%.

The analysis of carbonated beverages (e.g., ‘Coke’ or soda type) consumption allowed to conclude that Poles with lower than secondary education consume them most often (10.9% of the respondents). The equally high percentage was observed in the case of the respondents with secondary education 8.5% of the respondents, over twice more than in the case of the respondents with higher education (4.0%). Respondents with higher education consume the carbonated beverages of ‘Coke’ type less frequently.

Differences in the distribution of answers were also observed in relation to:

- still, fruit drinks– beverages of such type were consumed more frequently by respondents with lower secondary or secondary education (8.8%);
- carbonated, fruit drinks–most consumed by respondents with secondary (5.7%) and lower secondary education (4.6%). Consumers with higher education drink such beverages less frequently (2.0%);
- flavored waters, energy, and isotonic drinks, the statistical significance was confirmed by means of analyzing the education structure of the respondents using the chi-square independence test (Table 2). However, Figure 2 as well as Table 2 shows small differences resulting from differences in the level of education. Consumers with higher education drink flavored water significantly less frequently—in the case of comparing their everyday consumption, only 1.3% of the consumers with higher education declared that they drink them every day. For comparison, in the case of consumers who completed secondary education, the percentage was 8.0%, and with lower than education it amounted to 7.2%. Energy drinks are consumed more frequently by people with lower than secondary education (5.1% of the respondents) and decidedly less often by people with higher education (2.1% of the respondents). Analogically, the dependence was observed in the case of isotonic drinks. People with higher education drink such beverages less frequently (1.3%) than people with lower than secondary education (3.4%).

**Figure 3:**
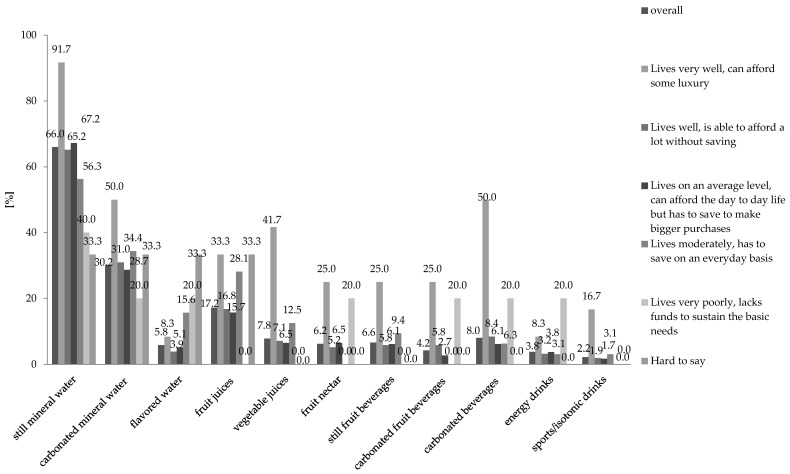
Beverages consumed on an everyday basis versus the financial situation of the respondents *n* = 500.

An attempt was made to describe the dependence between the financial situation of the Polish responders and the frequency of consumption of particular beverage types. The structure of the respondents’ group consists of almost 90% of people who declared that the “live well” or “on an average level” (Table 1). It would be a misuse to compare responses of groups, which are so uneven in number. The results that can be reported can be limited to some observations (Figure 3). Of all observations, the two following are the most numerical: 91.7% of the respondents who declared that their financial situation is good, stated that they drink still mineral water almost every day. Carbonated mineral water is most frequently consumed by people who are in a very good financial situation (50% of the respondents from this range).

Isotonic drinks are most often consumed by people who describe their financial situation as very good 16.7% of the respondents drink them every day. Such beverages are consumed relatively often (minimum once a week) by people who describe their financial situation as good 24.4%.

Statistical analysis carried out using Spearman’s rank correlation coefficient (Table 3) indicated that the frequency of consumption of selected drinks is related to each other (Table 3). That means, e.g., that the respondents who declared consumption of isotonic drinks also often drink energy drinks (r = 0.7085) and carbonated beverages of the ‘Coke’ type and carbonated fruit drinks (r = 0.7528). A weaker linear relationship, but also worth emphasizing, occurs in the case of frequency of consumption of fruit, carbonated beverages, and still, fruit beverages (r = 0.5775) or flavored waters (r = 0.5306).

## 4. Discussion

The results obtained within this study confirm and deepen the knowledge about the frequency of beverage consumption in correlation with sex, age, income, and education of the studied group of adults, who were of Polish nationality. It is indisputable that excess sugar in the diet affects the health of the consumers. Many times it was shown that consumption of regular, sugar-sweetened beverages increases the risk of becoming overweight or obese-related diseases [24,25,26]. The majority of Polish consumers (87.4%) declare that they consume added sugar [27]. Sugar-sweetened beverages are undoubtedly one of its sources. Overall, according to the research, when compared to adults from the USA or Spain, a smaller percentage of Polish adults reported consuming SSB at least once a day. However, individual consumption of SSB is very heterogeneous, and according to the results, the frequency of intake of beverages varied within sex, education, and age [28,29]. 

In contrast to SSB, water is crucial for, essentially, all body functions and most importantly for thermoregulation. According to the European Food Safety Authority (EFSA), water should be the main beverage in the diet [30]. What can be assessed/considered as positive in the behavior of Polish adults is that water is the most popular and most frequently consumed beverage among the participants in the study. The same was reported for the US population by Kuczmarski et al. [31]. To sum it up a statistically important dependency was observed between the gender of the consumer and the frequency of consumption of mineral water, noncarbonated fruit beverages, energetic and isotonic drinks. As confirmed for Mexican adults [32], also in Poland men consumed more sweetened beverages than women. However, it was not concluded that there is a dependency between the frequency of water consumption and the age of the consumer (statistically important dependency was observed between the age of the consumer and the frequency of consumption: flavored water, fruit nectars, carbonated fruit beverages, carbonated beverages and energy as well as isotonic drinks). It was suggested that there is a much higher percentage of obese and overweight men than women in certain parts of the world such as e.g., China, Germany, France [33]. In the light of the mentioned research it should be a warning light for dietitians and health professionals because in this study men declared to consume water and other non-carbonated beverages more rarely as opposed to carbonated beverages of the ‘Coke’ type, fruit drinks or carbonated water. What is more sugar-sweetened beverage intake may have an impact on reproductive parameters in young men [34]. Although the relationship between education and SSB consumption is statistically significant (Figure 2), the differences between the groups are slight. Among the nine beverages that do contain sugar, fruit juice decidedly comes first. However, due to calorific value (resulting from high fructose content), it should not be consumed in excess. Aggregating the consumption of all SSB could make a more compelling argument that they are contributing to obesity in Poland. Figure 2 shows that except for mineral waters, individual sweetened beverages are consumed every day by less than 19% of the respondents, but added together, they may be above 40%. Conversely, mineral water consumption could be promoted as non-caloric alternatives. This thought may motivate some people to consume tap water if it is potable and acceptable from a taste standpoint. Such high consumption of bottled beverages contributes to a high amount of glass or plastic containers polluting the environment if they are not recycled.

One of the main findings of this study indicates that consumption of sugar-sweetened beverages is higher among young adults as reported by Ozen et al. [35]. The fact that energy drinks are more often consumed by people under 45 years of age may be associated with the professional activity and general physical activity of this group of respondents. Therefore, these beverages are consumed less frequently by respondents in the 60+ group. 

As far as study limitations are concerned, there are a few worthy of mention. The first one is that it is based on self-reported data only, but anonymity and confidentiality were guaranteed to minimize the negative impact of such an approach. At the beginning of the questionnaire, the respondents were informed that the survey was anonymous and the results would be used for scientific purposes only. Another limitation of the study is the limited sample size. Making more observations would allow for more precise analysis, especially in the context of e.g., income. In our research, the respondents used personal judgment to respond to questions concerning income. A quantitative measure of income, which could be used in future research, should allow for more diverse responses.

It would be valuable to repeat this study to learn more about SSB consumption during autumn or wintertime or other periods of the Covid 19 lockdown. It is important to bear in mind that the level of restrictions varies among countries. Future research: based on the observations, made during the study we have concluded that further research should focus on checking the influence of introducing the sugar tax on the behaviors of Poles and comparing the level of SSB consumption in winter and summer

In the course of this study, it could not be determined if consumption of SSB was influenced by material status due to the lack of data. Block et al. mention that food choices are made on the basis of what is available to the consumer and due to limited resources, consumers who are worse off look for products that provide them with the most calories for the lowest price [36]. Another probable explanation is that material status affects our drinking habits, and these habits don’t change much later in life or at least in a short period of time e.g., a few years [37]. In the light of the introduction of “sugar taxes” in more and more countries [38], including Poland, the influence of this tax may have a progressive impact, which means that it will affect consumers with a lower material status than the ones who are in a better financial situation [39].

If we look at the relationship between the level of education and the frequency of consuming particular beverages we will observe an important statistical dependency. Frequent drinkers of flavored waters, energy drinks, and isotonic drinks are less educated. A similar correlation was observed among nationally representative samples of U.S. adults [37]. According to the results presented by Altman et al., public awareness of sugar taxes (and their public health impact) was higher among respondents with higher education levels [38]. Designing actions aimed at raising awareness and drawing their attention to the nutritional value of the purchased products is currently one of the trends in public health policies [40]. It was already ascertained that posters and signs attract consumers’ attention and provide useful information [41]. However, the fact that nutritional information is still competing with information such as pricing and brand names is an on-going problem. This may encourage consumers to choose ‘low-nutrient foods and beverages’ [42].

In general, the results correlate very well with data from the research by Zagorsky [37,43]. Firstly, the consumption of SSB tends to go down with age. However, in contrast to Zagursky, it also goes down with education. Secondly, a correlation between consumption of SSB and income could not be verified. Observations made in the US saw that people with low income consume more SSB [44]. Thirdly, adults drink SBB less often than younger consumers, and women drink them less often than men.

Additionally, it is worth underlining the correlation between the frequency of consumption of selected beverages. As it was determined above, consumers who prefer sweetened carbonated beverages also prefer other high sugar-abundant beverages. On the other hand, consumers who prefer vegetable juices (one of the healthiest caloric beverages) also drink fruit juices and nectars, which indicates a certain correlation of pro-health behaviors [45]. Even though the frequency of consuming vegetable juices in the following research is not high it may be considered as a practical means of increasing vegetable intake. Generally, it can be said that people who declare to consume beverages, which are generally considered to be unhealthy (with high sugar content) choose similar beverages as do the people who show a tendency for consuming healthier beverages (water, fruit, and vegetable juices).

There are a few limitations to this study. The first one is that it is based on self-reported data only, but anonymity and confidentiality were guaranteed to minimize the negative impact of such an approach. At the beginning of the questionnaire, the respondents were informed that the survey was anonymous and the results would be used for scientific purposes only. Another limitation of the study is the limited sample size. Making more observations would allow for more precise analysis, especially in the context of e.g., income. In our research, the respondents used personal judgment to respond to questions concerning income. A quantitative measure of income, which could be used in future research, should allow for more diverse responses It would be valuable to repeat this study to learn more about SSB consumption during autumn or wintertime or other periods of the Covid 19 lockdown. It is important to bear in mind that the level of restrictions varies among countries. Future research: based on the observations, made during the study we have concluded that further research should focus on checking the influence of introducing the sugar tax on the behaviors of Poles and comparing the level of SSB consumption in winter and summer.

## 5. Conclusions

The aim of this study was to determine the most popular and the most frequently consumed sweetened beverages among Polish consumers in correlation to sex, age, income, and education of the studied group of adults.

The carbonated beverages of the ‘Coke’ type, and carbonated fruit beverages, on an everyday basis or nearly everyday basis, are preferred by men rather than women. Energy drinks are most often consumed by respondents who belong to the 30–44 age group, with lower than secondary education (5.1% of the respondents) and decidedly less often by people with higher education (2.1%). Energy and isotonic drinks are most often consumed by people who describe their financial situation as very good (16.7% of the respondents drink them every day); or good (24.4% of respondents drink them once a week). Identification of the frequency of sugar-sweetened beverage intake indicates directions for further national or regional action against the spread of obesity. Moreover, attention should be drawn to young males who consume beverages abundant in sugar most often. The frequency of drinking still mineral water among Poles might motivate some people to use tap water if it is potable and acceptable from a taste standpoint. Environmental awareness of the consumers in the context of such a high amount of water consumption, which contributes to a large amount of unrecycled glass or plastic containers, might soon change the consumption patterns among the adult Poles.

Additionally, future research should consider the effect of seasons on the preferences regarding sweet beverages among adult Poles. The recent introduction of the sugar tax in Poland and the effects it has already had on the attitudes of Polish people towards sugar-sweetened beverages should also be discussed. What is more, the preferences might change because of the Covid-19, when some of the consumers started to live more responsibly and more concentrated on a healthier life.

## Figures and Tables

**Table 1:** General characteristics of participants.

		N	(%)
			
Gender	women	240	48.0
	men	260	52.0
			
Age	18 to 29	87	17.4
	30 to 44	149	29.8
	45 to 59	116	23.2
	over 60	148	29.6
			
Education	lower than secondary	175	35.0
	secondary	176	35.2
	high	149	29.8
			
Material status	lives very well, can afford some luxury	12	2.4
	lives well, is able to afford a lot without saving	155	31.0
	lives on an average level, can afford the day to day life but has to save to make bigger purchases	293	58.6
	lives moderately, has to save on an everyday basis	32	6.4
	lives very poorly, lacks funds to sustain the basic needs	5	1.0
	hard to say	3	0.6

**Table 2:** Consumption of beverages with confirmed statistical significance taking into account the educational structure *n* = 500.

	Flavoured Water (*p*-Value 0.002) [%]			Energy Drinks (*p*-Value 0.045) [%]			Isotonic Drinks (*p*-Value 0.003) [%]		
Education	Lower than Secondary	Secondary	High	Lower than Secondary	Secondary	High	Lower than Secondary	Secondary	High
Every day or almost every day	7.4	8.0	1.3	5.1	4.0	2.0	3.4	1.7	1.3
3–4 times a week	17.7	11.9	12.1	10.3	2.8	4.7	4.0	6.8	2.7
1–2 a week	13.7	21.6	24.8	14.3	11.9	10.7	11.4	6.8	10.1
Few times a month	23.4	13.1	20.1	13.7	14.8	16.1	14.3	11.4	13.4
Sporadically (occasionally. less than once a month)	22.3	34.1	30.9	28.0	30.1	39.6	40.0	26.7	43.0
Never	15.4	11.4	10.7	28.6	36.4	26.8	26.9	46.6	29.5

**Table 3:** Correlation of Selected Drinks consumed by Polish Adults, using Spearman’s rank correlation coefficient.

	1	2	3	4	5	6	7	8	9	10	11
1 ^a^			0.1128	0.2903	0.2399	0.2103	0.1349	0.1004	0.0877		0.1180
2			0.3052	0.2045	0.2449	0.2344	0.2037	0.3475	0.3444	0.1993	0.2090
3				0.4577	0.3772	0.4650	0.5615	0.5306	0.4484	0.3785	0.3368
4					0.4923	0.5472	0.5163	0.3960	0.3817	0.2951	0.2562
5						0.4676	0.3287	0.3268	0.2365	0.3322	0.3958
6							0.5920	0.4725	0.4178	0.3937	0.3613
7								0.5775	0.48148	0.4013	0.3436
8									0.7528	0.5113	0.4089
9										0.5288	0.4365
10											0.7085
11											

^a^ 1 still mineral water, 2 carbonated mineral water, 3 flavored water, 4 fruit juices, 5 vegetable juices, 6 fruit nectar, 7 still fruit beverages, 8 carbonated fruit beverages, 9 carbonated beverages e.g., coke or soda, 10 energy drinks, 11 sports/isotonic drinks.

## Data Availability

The data presented in this study are available on request from the corresponding author. The data are not publicly available due to ongoing investigations.

## Supplementary Materials

The following are available online at https://www.mdpi.com/article/10.3390/ijerph18137029/s1, Table S1: Differences in the frequency of consumption of particular beverages depending on the gender, Table S2: Differences in frequency of consumption of particular beverages depending on age *n* = 500.
