# Supplementary material for: Assessment of the Frequency of Sweetened Beverages Consumption among Adults in Poland
Source: Int J Environ Res Public Health. 2021 Jun 30;18(13):7029. doi: 10.3390/ijerph18137029 (PMC8296859; doi:10.3390/ijerph18137029)
Supplement: Supplementary file 1 [file ijerph-18-07029-s001.zip › ijerph-1235716-supplementary.pdf]

Table S1. Differences in the frequency of consumption of particular beverages depending on the gender.

	Generally	Woman	Man
		[%]	
1.01. still mineral water (chi-square test; p= 0.027)			
Every day or almost every day	66.0	73.3	59.2
3-4 times a week	11.6	9.2	13.8
1-2 times a week	8.8	6.3	11.2
Few Times a month	5.6	3.8	7.3
Sporadically (occasionally, less than once a month)	6.4	6.3	6.5
Never	1.6	1.3	1.9
1.02. carbonated mineral water (chi-square test; p= 0.390)			
Every day or almost every day	30.2	28.3	31.9
3-4 times a week	18.6	17.1	20.0
1-2 times a week	15.4	14.6	16.2
A few times a month	12.0	13.3	10.8
Sporadically (occasionally, less than once a month)	16.8	17.5	16.2
Never	7.0	9.2	5.0
1.03. flavored water (chi-square test; p= 0.373)			
Every day or almost every day	5.8	5.4	6.2
3-4 times a week	14.0	13.8	14.2
1-2 times a week	19.8	20.8	18.8
Few times a month	18.8	15.0	22.3
Sporadically (occasionally, less than once a month)	29.0	30.8	27.3
Never	12.6	14.2	11.2
1.04. fruit juices (chi-square test; p= 0.342)			
Every day or almost every day	17.2	20.8	13.8
3-4 times a week	21.4	21.3	21.5
1-2 times a week	26.0	24.6	27.3
Few Times a month	23.8	21.3	26.2
Sporadically (occasionally, less than once a month)	11.0	11.3	10.8
Never	0.6	0.8	0.4
1.05. vegetable juices (chi-square test; p= 0.054)			
Every day or almost every day	7.8	8.8	6.9
3-4 times a week	10.6	14.2	7.3
1-2 times a week	17.4	19.6	15.4
Few times a month	21.8	18.8	24.6
Sporadically (occasionally, less than once a month)	30.6	27.5	33.5
Never	11.8	11.3	12.3
1.06. fruit nectar (chi-square test; p= 0.107)			
Every day or almost every day	6.2	6.7	5.8
3-4 times a week	9.6	12.9	6.5
1-2 times a week	18.4	20.4	16.5
Few times a month	27.0	23.8	30.0
Sporadically (occasionally, less than once a month)	31.2	29.2	33.1
Never	7.6	7.1	8.1

	**Generally**	**Woman**	**Man**
		[%]	
1.07. still fruit beverages (chi-square test; p= 0.017)			
Answer	Generally	Woman	Man
Every day or almost every day	6.6	7.5	5.8
3-4 times a week	12.8	12.5	13.1
1-2 times a week	18.8	18.8	18.8
Few times a month	25.0	18.3	31.2
Sporadically (occasionally, less than once a month)	28.8	32.9	25.0
Never	8.0	10.0	6.2
1.08. carbonated fruit beverages (chi-square test; p= 0.075)			
Every day or almost every day	4.2	3.3	5.0
3-4 times a week	13.8	11.7	15.8
1-2 times a week	17.6	17.5	17.7
Few times a month	25.4	22.9	27.7
Sporadically (occasionally, less than once a month)	29.2	31.3	27.3
Never	9.8	13.3	6.5
1.09. carbonated beverages coke and soda type (chi-square test; p= 0.103)			
Every day or almost every day	8.0	5.8	10.0
3-4 times a week	15.2	13.8	16.5
1-2 times a week	17.8	16.7	18.8
Few times a month	25.2	24.6	25.8
Sporadically (occasionally, less than once a month)	26.0	28.8	23.5
Never	7.8	10.4	5.4
1.10. Energy drinks (chi-square test; p= 0.034)			
Every day or almost every day	3.8	2.9	4.6
3-4 times a week	6.0	3.3	8.5
1-2 times a week	12.4	12.1	12.7
Few times a month	14.8	13.8	15.8
Sporadically (occasionally, less than once a month)	32.2	31.3	33.1
Never	30.8	36.7	25.4
1.11. sports/isotonic drinks (chi-square test; p= 0.006)			
Every day or almost every day	2.2	2.1	2.3
3-4 times a week	4.6	4.2	5.0
1-2 times a week	9.4	8.8	10.0
Few times a month	13.0	10.4	15.4
Sporadically (occasionally, less than once a month)	36.2	31.3	40.8
Never	34.6	43.3	26.5

Table S2. Differences in frequency of consumption of particular beverages depending on age n=500

	generally	18-29 y/o	30-44 y/o	45-59 y/o	60+ y/o
	[%]				
1.01. still mineral water (chi-square test; p = 0.884)					
Every day or almost every day	66.0	67.8	66.4	65.5	64.9
3-4 times a week	11.6	16.1	11.4	12.1	8.8
1-2 times a week	8.8	6.9	8.1	6.9	12.2
A few times a month	5.6	4.6	6.0	6.0	5.4
Sporadically (ocassionally. less than once a month)	6.4	2.3	6.7	7.8	7.4
Never	1.6	2.3	1.3	1.7	1.4
1.02. carbonated mineral water (chi-square test; p= 0.260)					
Every day or almost every day	30.2	31.0	30.2	34.5	26.4
3-4 times a week	18.6	11.5	22.1	19.0	18.9
1-2 times a week	15.4	17.2	19.5	12.9	12.2
Few times a week	12.0	16.1	10.1	9.5	13.5
Sporadically (ocassioanlly. less than once a week)	16.8	18.4	13.4	19.0	17.6
Never	7.0	5.7	4.7	5.2	11.5
1.03. flavored water (chi-square test; p=0.001)					
Every day or almost every day	5.8	5.7	4.7	5.2	7.4
3-4 times a week	14.0	13.8	16.1	10.3	14.9
1-2 times a week	19.8	21.8	24.2	21.6	12.8
Few times a month	18.8	24.1	16.1	18.1	18.9
Sporadically (ocassionally. less than once a month)	29.0	24.1	34.2	33.6	23.0
Never	12.6	10.3	4.7	11.2	23.0
1.04. fruit juices (chi-square test; p= 0,129)					
Every day or almost every day	17.2	19.5	14.8	18.1	17.6
3-4 times a week	21.4	21.8	22.1	16.4	24.3
1-2 times a week	26.0	33.3	26.8	31.0	16.9
Few times a month	23.8	18.4	24.2	24.1	26.4
Sporadically(ocassionally. less than once a month)	11.0	6.9	12.1	10.3	12.8
Never	0.6	0.0	0.0	0.0	2.0
1.05. vegetable juices (chi-square test; p= 0.278)					
Every day or almost every day	7.8	9.2	6.0	9.5	7.4
3-4 times a week	10.6	11.5	12.1	11.2	8.1
1-2 times a week	17.4	19.5	24.2	14.7	11.5
Few Times a month	21.8	16.1	20.8	20.7	27.0
Sporadically (ocassionally. less often than once a month)	30.6	27.6	28.9	32.8	32.4

	generally	18-29 y/o	30-44 y/o	45-59 y/o	60+ y/o
			[%]		
Never	11.8	16.1	8.1	11.2	13.5
1.06. fruit nectars (chi-square test; p= 0.044)					
3-4 times a week	9.6	19.5	8.7	5.2	8.1
1-2 times a week	18.4	18.4	22.1	16.4	16.2
Few times a month	27.0	27.6	24.2	31.9	25.7
Sporadically (ocassionally. less than once a month)	31.2	25.3	30.9	34.5	32.4
Never	7.6	2.3	8.1	5.2	12.2
1.07. still fruit beverages (chi-square test; p= 0.181)					
Every day or almost every day	6.6	5.7	6.0	6.9	7.4
3-4 times a week	12.8	14.9	14.1	8.6	13.5
1-2 times a week	18.8	24.1	17.4	21.6	14.9
Few times a week	25.0	27.6	22.8	29.3	22.3
Sporadically (ocassionally. less than once a month)	28.8	23.0	32.9	29.3	27.7
Never	8.0	4.6	6.7	4.3	14.2
1.08. carbonated fruit beverages (chi-square test; p= 0.013)					
Every day or almost every day	4.2	6.9	5.4	2.6	2.7
3-4 times a week	13.8	19.5	12.8	13.8	11.5
1-2 times a week	17.6	18.4	24.2	12.1	14.9
Few times a month	25.4	23.0	27.5	28.4	22.3
Sporadically (occasionally. less than once a month)	29.2	28.7	24.2	32.8	31.8
Never	9.8	3.4	6.0	10.3	16.9
1.09. carbonated beverages of the coke and soda type (chi-square test; p= 0.034)					
Every day or almost every day	8.0	9.2	10.1	5.2	7.4
3-4 times a week	15.2	25.3	16.1	8.6	13.5
1-2 times a week	17.8	17.2	22.1	16.4	14.9
Few times a month	25.2	23.0	26.2	28.4	23.0
Sporadically (occasionally. less than once a month)	26.0	21.8	19.5	32.8	29.7
Never	7.8	3.4	6.0	8.6	11.5
1.10. energy drinks (chi-square test; p= 0.023)					
Every day or almost every day	3.8	3.4	5.4	1.7	4.1
3-4 times a week	6.0	9.2	6.0	5.2	4.7
1-2 times a week	12.4	14.9	15.4	6.9	12.2
Few times a month	14.8	13.8	19.5	12.1	12.8
Sporadically (occasionally. less than once a month)	32.2	31.0	35.6	35.3	27.0
Never	30.8	27.6	18.1	38.8	39.2
1.11. isotonic drinks (chi-square test; p= 0.003)					
Every day or almost every day	2.2	1.1	2.0	1.7	3.4
3-4 times a week	4.6	6.9	6.0	3.4	2.7
1-2 times a week	9.4	8.0	15.4	5.2	7.4
Few times a month	13.0	19.5	13.4	12.1	9.5

	generally	18-29 y/o	30-44 y/o	45-59 y/o	60+ y/o
			[%]		
Sporadically (occasionally less than once a month)	36.2	36.8	40.3	29.3	37.2
Never	34.6	27.6	22.8	48.3	39.9
